# National Health Insurance unpacked: Part 2: Accreditation of primary care facilities

**DOI:** 10.4102/safp.v62i1.5140

**Published:** 2020-06-08

**Authors:** Bob Mash

**Affiliations:** 1 Division of Family Medicine and Primary Care, Faculty of Medicine and Health Sciences, University of Stellenbosch, Cape Town, South Africa

## Introduction

This four-part series in the *South African Family Practice* journal unpacks the details of National Health Insurance (NHI) as proposed in the NHI Bill that went before Parliament in July 2019.^1^ In Part 2 of the series, we look at the accreditation of primary care facilities under NHI.

## What is the intended package of care?

One of the requests from family medicine practitioners is for clarity on the intended package for primary care services. The package will clearly be a comprehensive one about covering the whole lifecycle from conception to end-of-life care. It will also be comprehensive about including health promotion, disease prevention, treatment, palliative care and rehabilitation, as appropriate to primary care. A comprehensive package should also cover the entire range of presentations and burden of disease in South Africa with services appropriate to the primary care level. Acute, chronic and emergency conditions would all need to be included.

A community-orientated primary care approach is already implemented in South Africa, particularly in poorer and more vulnerable communities.^2,3^ This means that primary care is delivered by a combination of facility-based and community-based services which also integrate a public health and primary care approach. Community-based services include teams of community health workers working with professional nurses (ward-based outreach teams) who are responsible for delineated geographic areas and specific households.

Quality primary care also implies a commitment to ensuring access to care for those patients registered with the practice, and striving for a person-centred approach, continuity and coordination of care.

Services would need to be provided by competent and motivated primary care providers. Services might all be provided by one facility, or by a network of facilities in the local area, to enable a comprehensive package. Providers would include doctors (family physicians, medical officers and general practitioners), nurse practitioners, professional nurses and community health workers. A range of administrative and support personnel will also be needed, such as receptionists, clerks and facility managers. A range of additional health professionals would also be required to ensure the minimum package, such as pharmacists, dieticians, physiotherapists, occupational therapists, speech therapists, specialised nurses and counsellors. These additional health professionals would most likely be located at community health centres. Access to social services and social workers will also be needed.

The National Department of Health has not yet defined a more specific list of services that would be required. The Minister will gazette the benefits on recommendation by an advisory committee, that can only be established once the Bill is enacted.

## How will gatekeeping and referral to the next level of care work?

One of the requirements for patient benefits to be paid by the NHI is that they must first access health care services at the primary care level and must adhere to the prescribed referral pathway. Primary care, therefore, will be the gate to the rest of the health system under the NHI and primary care providers, hence the gatekeepers. Patients will, of course, be able to bypass primary care for certain emergencies that require immediate hospital care. As everyone will be registered with a primary care facility with a unique patient identifier, it should become possible for their electronic record to be accessible at the hospital. This will improve coordination of care between levels of the health system.

## What standards will be used to accredit practices?

Practices must meet a number of criteria, as specified in the Bill, in order to be accredited by the Fund:

- Certification by the Office of Health Standards Compliance
- Registration of health professionals with the appropriate Council
- Provide the minimum package of primary care
- Have the necessary mix and number of health professionals to deliver the package
- Adhere to the relevant clinical protocols and guidelines
- Adhere to the referral pathways
- Submit the necessary data to the health information system
- Adhere to the national pricing regimen for services.

The Office of Health Standards Compliance must certify the practice as meeting the required standards. The National Department of Health has developed criteria for the ideal primary care clinic and these will form the basis of certification by the Office of Health Standards Compliance. There are currently 10 components and 32 sub-components, as shown in Table 1, that need to be measured.^4^

**TABLE 1:** Framework of the ideal clinic criteria.

Components	Sub-components
Administration	1. aSignage and notices 2. Staff identity and dress code 3. Patient service organisation 4. Management of patient records
Integrated clinical service management	5. Clinical service provision 6. Access to medical, mental health and allied health professionals 7. Management of patient appointments 8. Coordination of primary healthcare services 9. Clinical guidelines and protocols 10. Infection prevention and control 11. Patient waiting times 12. Patient experience of care
Medicines, supplies and laboratory services	13. Medicines and supplies 14. Management of laboratory services
Human resources for health	15. Staff allocation and use 16. Professional standards, performance management and development system
Support services	17. Finance and supply chain management 18. Hygiene and cleanliness 19. Security 20. Outbreak disaster preparedness
Infrastructure	21. Physical space and routine maintenance 22. Essential equipment and furniture 23. Bulk supplies 24. Information communication technology
Health information management	25. District health information system
Communication	26. Internal communication 27. Community engagement
District health system support	28. District health system support 29. Emergency response 30. Referral system
Implementing partners and stakeholders	31. Implementing partners support 32. Multi-sectoral collaboration

*Source*: National Department of Health. Ideal Clinic Definitions, Components and Checklists [homepage on the Internet]. 2018 [cited 2020 May 05]. Available from: https://www.idealhealthfacility.org.za/^4^

## What is a contracting unit for primary health care?

The district health system in South Africa divides the country into 44 contiguous health districts and eight metropolitan areas. Contracting units for primary health care (CUP) will be established around the 260 district hospitals and their primary healthcare platforms. The CUP will be responsible for accrediting the primary health care services, such as clinics, health centres, general practitioner practices and ward-based outreach teams. The CUP will not be responsible for accrediting the district hospital.

The CUP will identify local service needs in terms of demographics and epidemiological profiles. The CUP will also identify and accredit local primary care facilities to meet these needs. These facilities are referred to as health establishments in the Bill. The CUP will contract with primary care establishments, in either the public or private sectors, to provide services and disperse NHI funds according to the contract. The contract will specify the required access to, range and volume of services, as well as the expected performance, quality requirements and data to be collected on the NHI digital health information system.

Some aspects of the CUPs are not finalised. For example, it is not yet decided who the juristic entity will be that CUPs should contract with. It could be the providers themselves or the establishment as an organisation. It is also not yet clear if the CUPs will take responsibility for the supply chain (e.g. medication, laboratory services, consumables) to the primary care establishments.

In addition, the CUP will be responsible for accessing information on the health needs of the community, improving access to health services for the local population, and ensuring that the referral and transport system is functional. They will also be responsible for resolving complaints about the health services.

## What happens if you don’t meet the standards?

The CUP can only contract with providers that are certified by the Office of Health Standards Compliance as meeting the required standards and with accredited establishments. Accreditation will be reviewed every 5 years and, if the primary care facility is not able to meet the criteria, then a loss of accreditation will lead to a loss of the ability to contract with the CUP.

## What kind of ongoing quality assurance will be required?

The CUP is responsible for the ongoing collection of health information on the range, volume and quality of services provided. The District Health Management Team will be responsible for monitoring and improving quality and patient safety.
